# Surgical leadership – a plea for action in rapidly changing times

**DOI:** 10.1515/iss-2019-0009

**Published:** 2019-05-04

**Authors:** Joachim Jähne

**Affiliations:** Clinic for General and Digestive Surgery, Center for Endocrine, Oncological and Metabolic Surgery, DIAKOVERE Henriettenstift, Marienstrasse 72-90, D-30171 Hannover, Germany

Currently, we face multiple, sometimes even dramatic changes worldwide. Not only in the Western hemisphere, but also in emerging countries there are increasing trends towards nationalism, isolation and delimitation. Social media demonstrate self-optimization and it can be hardly denied, that apparently the “Me” is above all.

These political and social changes are in sharp contrast to the reality of working conditions in medicine in general and in surgery in particular. Treating patients with the aspiration of the best available therapy has been part of teamwork since ancient history but has emerged even more in the last years. Multimodal treatment in mutliprofessional and interdisciplinary teams is the current standard. Realizing these facts, we also face a shortage of young medical students applying for a surgical career, increased demands for an intact work-life balance as well as altered family structures with more women being fulltime employed. This professional environment requires changed organizational structures and improved abilities and skills to manage a surgical department and to act as a leader in team structures. Simply said – it is not enough to be an excellent surgeon. Economic knowledge and non-technical skills matter as well [1]. And what else is required to be a good surgical leader?

The current issue of *Innovative Surgical Sciences* (*ISS*) aims to highlight the essentials of modern surgical leadership in different countries and cultures. One important issue in surgical leadership is pointed out by Ohki in his paper: you need to guide surgeons by getting them excited about the work, by creating friendships within the team and by instilling a feeling of secureness. The experiences from Japan clearly demonstrate that non-technical skills are much more important for an inspiring working environment than, for example, financial incentives. It also shows that social activities outside the hospital add substantial value to the team spirit.

Wallner and Solecki from Poland, in their paper, argue for a structured learning course within a system of harmonized, methodologically correct system of surgical training and education. This is most likely a good concept, because it is well accepted that a brilliant surgeon is not necessarily an excellent surgical leader as well. Therefore, a structured learning system of non-technical skills may be helpful to increase leadership abilities.

As regards the situation in Germany, Schmitz-Rixen and Grundmann show that economic knowledge is crucial in leading a surgical department. In many German hospitals, leadership by objectives is very common. These objectives, however, refer to case numbers and case severity, but they do not reflect the soft skills of leadership. Additionally, it is very important to offer structured surgical training programs to fulfil the needs of the younger generation as well as women. The paper from Germany addresses one other increasingly important issue: we face more and more colleagues with signs of distress, depression and burnout. This could be a topic for a separate special issue of *ISS*, and it clearly demonstrates that surgical leaders need to have a close and critical look to their staff in order to recognize these symptoms of burnout in surgeons to offer help and relief.

In contrast to the situation in Japan and Europe, the paper from Ghana points out, that surgical leadership necessitates becoming politically involved to improve the surgical care of the people. Africa faces substantial infrastructural challenges, low manufacturing capacity and a significant lack of political will to change the situation of surgical patients. It is noteworthy, that surgical disease has a higher mortality than the politically more highly-ranked fight against HIV/AIDS and tuberculosis. Frimpong-Boateng and his co-author Frank Edwin impressively describe that surgical leaders in Africa need to engage with political authorities and to advocate for surgical care in order to obtain political priority. These views on surgical leadership are in complete contrast to the challenges in first-world countries, but there is one issue which unifies the various aspects – the development of information and communication technology. The paper from Ghana illustrates the possible benefits of digitalization in surgery and clearly this will most likely improve the possibilities of surgery in the future for the benefit of our patients worldwide.

A different, but very relevant aspect of surgical leadership is stressed by John Primrose from Great Britain. The British paper shows that leadership is expected when treating patients and that patients suffer when leadership is missing. Leadership is mentorship for younger surgeons using inspiration and motivation. It is also shown that true leadership in the context of scientific surgical societies results in rationalization and centralization for the benefit of the patients operated on in highly specialized institutions. On the other hand, Primrose argues for a consolidation of the huge amount of surgical societies, a challenge that is faced in many other countries as well.

The perspective of the American College of Surgeons (ACS), presented by Barbara Bass, shows how such a consolidation could work. The ACS functions as an umbrella organization for all surgical disciplines and provides a wide range of offers to establish surgical leadership. Changes in the surgical environment as well as the increasing diversity of the new members in the surgical profession are welcome to create the societies of the future. By various programs, by the fellowships as well as by training courses the ACS tries to help its members to become surgical leaders in a more and more complex professional world. There are hardly any other surgical societies worldwide which offer such a wide range of activities to improve the knowledge about and the abilities of surgical leadership.

Surgical leadership today and in particular in the future does not only mean to be a technically well-trained surgeon. It means

– improvement in non-technical skills and in particular communication to inspire staff to obtain the best possible results
– promotion of diversity in gender, ethnic background and age to get the best available decisions [2], [3]
– involvement not only in the hospital and in the scientific community, but also in political institutions to raise awareness of surgical disease and surgical therapy
– serving the people you are working with to improve their skills and abilities
– cooperation instead of confrontation. Every time people work together the result is usually better than in stand-alone solutions.

Taking these and various other, not yet mentioned issues about surgical leadership like charisma, personal integrity and honesty into account it should be possible to shape the future of our fascinating discipline. Yet, we need to act. Leadership is nothing that comes from itself – we need the will to lead.
